# Systemic inhibition of myeloid dendritic cells by circulating HLA class I molecules in HIV-1 infection

**DOI:** 10.1186/1742-4690-9-11

**Published:** 2012-01-30

**Authors:** Jinghe Huang, Maha Al-Mozaini, Jerome Rogich, Mary F Carrington, Katherine Seiss, Florencia Pereyra, Mathias Lichterfeld, Xu G Yu

**Affiliations:** 1 Ragon Institute of MGH, MIT and Harvard, Boston, MA, USA; 2 Infectious Disease Division, Massachusetts General Hospital, Boston, MA, USA; 3 King Faisal Specialist Hospital & Research Center, Riyadh, Saudi Arabia

**Keywords:** HIV-1, dendritic cells, HLA, immunoregulation, Leukocyte Immunoglobulin Like Receptor (LILR)

## Abstract

**Background:**

HIV-1 infection is associated with profound dysfunction of myeloid dendritic cells, for reasons that remain ill-defined. Soluble HLA class I molecules can have important inhibitory effects on T cells and NK cells, but may also contribute to reduced functional properties of professional antigen-presenting cells. Here, we investigated the expression of soluble HLA class I isoforms during HIV-1 infection and assessed their functional impact on antigen-presenting characteristics of dendritic cells.

**Results:**

Soluble HLA class I molecules were highly upregulated in progressive HIV-1 infection as determined by quantitative Western blots. This was associated with strong increases of intracellular expression of HLA class I isoforms in dendritic cells and monocytes. Using mixed lymphocyte reactions, we found that soluble HLA class I molecules effectively inhibited the antigen-presenting properties of dendritic cells, however, there was no significant influence of HLA class I molecules on the cytokine-secretion properties of these cells. The immunomodulatory effects of soluble HLA class I molecules were mediated by interactions with inhibitory myelomonocytic MHC class I receptors from the Leukocyte Immunoglobulin Like Receptor (LILR) family.

**Conclusions:**

During progressive HIV-1 infection, soluble HLA class I molecules can contribute to systemic immune dysfunction by inhibiting the antigen-presenting properties of myeloid dendritic cells through interactions with inhibitory myelomonocytic HLA class I receptors.

## Background

HIV-1 infection leads to massive immune activation that results from direct stimulation of immune cells by HIV-1 antigens, the release of large amounts of pro-inflammatory cytokines, and the systemic circulation of bacterial polysaccharide antigens after translocation from intestinal mucosal tissues [1]. This immune activation can cause counter-regulatory activities of inhibitory components of the immune system, such as increased recruitment of regulatory T cells [2], upregulation of inhibitory receptors on antigen-specific T cells [3,4], and enhanced expression of immunoregulatory receptors on dendritic cells [5,6]. These mechanisms may in part protect the host against immune pathology by limiting over activation of the immune system, but might also contribute to viral persistence by propagating immune dysfunction. Identifying immunomodulatory mechanisms that contribute to this functional disarray between stimulatory and inhibitory immunological pathways is an important step in understanding the pathogenesis of HIV-1 infection.

HLA class I isoforms are heterodimeric molecules that consist of a 44-kDa polymorphic glycoprotein (α chain) that is noncovalently associated with the 12-kDa non-polymorphic β2-microglobulin. These molecules are expressed on the surface of all human cells and have important functions for presenting antigenic peptides, and for priming and maintaining T cell immune responses. In addition, HLA class I molecules can also occur as soluble agents in the serum or plasma [7,8]. These soluble HLA class I molecules can either occur as intact, 44-kDa HLA class I heavy chains, or as 39-kDa variants that do not contain a transmembrane domain and result from alternative splicing [9]. 35-kDa species of soluble HLA class I isoforms have also been described and most likely represent proteolytic breakdown products of intact HLA class I heavy chains [10]. These soluble HLA molecules can have important systemic immunoregulatory effects by influencing survival and apoptosis of antigen-specific T cells and NK cells through interactions with receptors from the KIR or the C-type lectin family [11-13]. Leukocyte Immunoglobulin Like Receptors (LILR) represent an alternative group of HLA class I receptors that are predominantly expressed on dendritic cells and monocytes, which, as professional antigen-presenting cells, have central roles for generating adaptive immune responses and regulating immune activation through cytokine secretion [14]. Upon triggering by HLA molecules, these receptors can influence functional properties of professional antigen-presenting cells in an inhibitory or stimulatory fashion, and in this way importantly influence pathogen-specific immune defense mechanisms. During HIV-1 infection, the quantity of soluble HLA class I isoforms is increased in the plasma [15], but how these molecules can specifically affect the functional characteristics of circulating dendritic cells during HIV-1 infection is unclear at present. In this study, we show that soluble HLA class I isoforms can importantly alter the antigen-presenting properties of dendritic cells through interactions with the inhibitory myelomonocytic MHC class I receptors. These data indicate a previously unrecognized immunoregulatory pathway that contributes to immune dysfunction of dendritic cells in HIV-1 infection.

## Results

To investigate immunoregulatory effects of soluble HLA class I isoforms in HIV-1 infection, we initially focused on quantifying the expression of HLA class I molecules in plasma and PBMC. For this purpose, a group of patients with chronic progressive HIV-1 infection (n = 14, mean viral load of 28,484 (8,104-449,000) copies/ml, mean CD4 cell counts of 412.5 (195-1000) cells/ul), a cohort of HIV-1 elite controllers (n = 14, viral load < 75 copies/ml, mean CD4 cell counts of 630 (188-1134) cells/μl) and a background population of HIV-1 negative persons (n = 16), were recruited.

**Figure 1:**
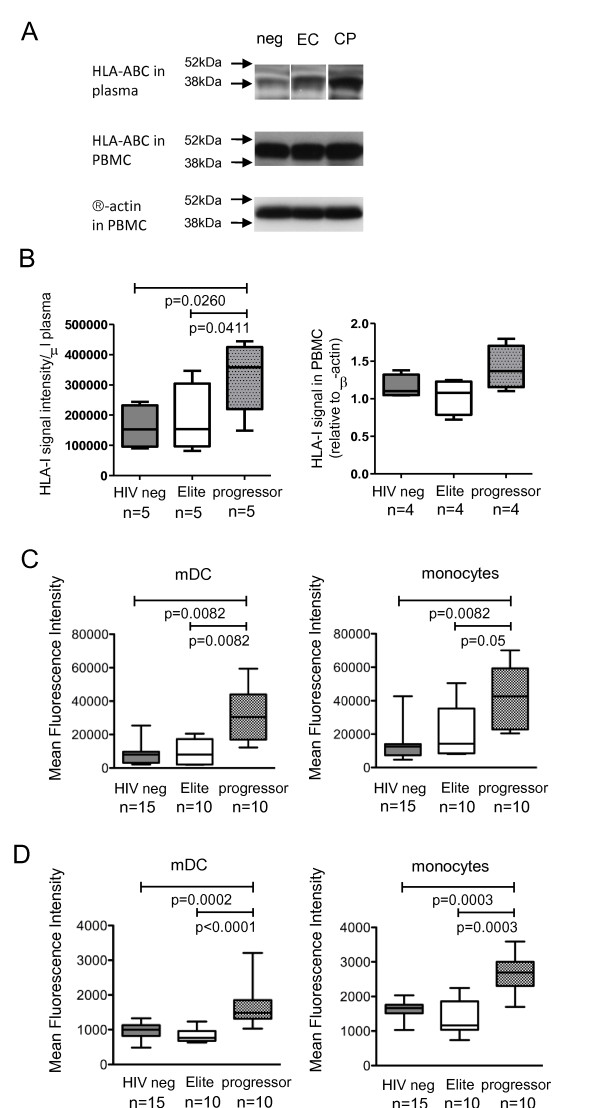
**Increased expression of soluble HLA class I molecules in progressive HIV-1 infection**. (A-B): Western blots reflecting HLA class I expression in the plasma and in PBMC of persons with progressive HIV-1 infection, elite controllers and HIV-1 negative individuals. (A) shows representative data from a given patient from each study cohort; (B) demonstrates cumulative data from indicated patient numbers. (C-D): Expression of intracellular (C) and membrane-bound (D) HLA class I molecules in monocytes and dendritic cells from the three study cohorts.

Western blot experiments indicated the presence of an HLA class I-specific, 44-kDa band in PBMC in study subjects from all three patient cohorts (Figure 1A/B). In contrast, Western blot experiments from plasma revealed a 39-kDa band of HLA class I molecules, which most likely represents splice variants that are secreted by cells as soluble molecules and lack a transmembrane domain [9]. These soluble HLA class I forms were significantly more strongly expressed in HIV-1 progressors than in elite controllers or HIV-1 negative persons (Figure 1A/B). HLA class I associated β2-microglobulin was also more strongly expressed in plasma of progressors as compared to elite controllers or HIV-1 negative persons (Additional File 1).

Since increased levels of soluble HLA class I molecules in progressors may result from secretion by peripheral blood leukcoytes, we assessed intracellular expression of HLA class I molecules in different subsets of PBMC from our study cohorts. We observed a significant upregulation of intracellular HLA class I molecules in monocytes and dendritic cells from progressors (Figure 1C). Surface expression of HLA class I molecules on dendritic cells and monocytes was also higher in progressors than in the reference cohorts, but these differences were less pronounced than intracellular HLA class I expression (Figure 1D). Despite the fact that HIV-1 is known to downregulate HLA class I expression [16], we did not observe differences between the surface or intracellular expression of HLA class I molecules on T cells from the different study cohorts (data not shown), likely because the proportion of HIV-1 infected cells among all T lymphocytes *in vivo *is very low. Overall, these data suggest that secretion of soluble HLA class I molecules from dendritic cells and monocytes contributes to higher levels of soluble plasma HLA class I isoforms during progressive HIV-1 infection.

To investigate functional effects of soluble MHC class I isoforms, we focused on how these molecules can affect the stimulatory characteristics of myeloid dendritic cells which have key roles for the generation of adaptive immune responses. For this purpose, we tested the antigen-presenting properties of monocyte-derived dendritic cells (MDDC) in the presence of plasma from three study cohorts, using mixed lymphocyte reactions. Briefly, MDDC derived from identical donors were incubated for 30 minutes with respective plasma samples, washed aggressively, and co-cultured with CFSE-labeled allogeneic T cells from an HIV-1 negative study subject. Using flow cytometry assays, > 99% of the MDDC expressed the dendritic cell surface markers HLA-DR and CD11c, and HLA class I expression was detectable on > 98% of these cells (Additional File 2). In comparison to MDDC treated with plasma from HIV-1 negative persons or elite controllers, MDDC exposed to plasma from HIV-1 progressors had significantly reduced abilities to expand allogeneic T cells; this was true both for allogeneic CD4 and CD8 T cell responses.

**Figure 2:**
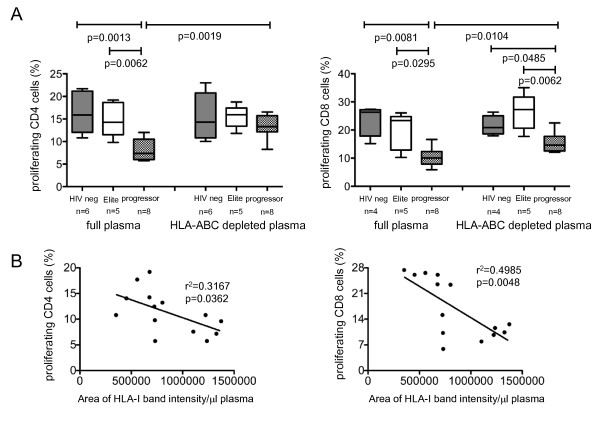
**Soluble HLA class I molecules inhibit antigen-presenting properties of MDDC**. (A-B): Soluble HLA class I molecules block allostimulatory activities of MDDC. MDDC were stimulated with plasma from HIV-1 progressors, elite controllers or HIV-1 negative persons with or without prior depletion of soluble HLA class I molecules. Subsequently, MDDC were mixed with allogeneic T cells. Data reflect proportions of proliferative allogeneic T cells (A) and correlations between proliferative allogeneic T cell responses and corresponding quantities of soluble HLA class I isoforms in the plasma (B).

To determine which plasma components in progressors inhibit the stimulatory properties of MDDC, we used monoclonal antibodies to deplete HLA class I isoforms from the plasma; this procedure selectively removed approximately 90% of human MHC class I molecules (Additional File 3). Moreover, expression of membrane-bound HLA class I molecules on MDDC was not affected by this procedure, and therefore did not influence the experimental outcome. These experiments showed that MDDC exposed to HLA class I-devoid plasma from progressors had significantly higher stimulatory properties compared to MDDC exposed to HLA-class I containing plasma from progressors. In contrast, no difference was observed in MDDC function following exposure to HLA class I-deficient vs. HLA class I-proficient plasma from HIV-1 elite controllers or HIV-1 negative persons (Figure 2A). Moreover, differences between allogeneic CD4 T cell proliferation were no longer visible after stimulation with MDDC exposed to HLA class I-depleted plasma form the three study cohorts (Figure 2A). We also observed a strong inverse correlation between the proliferative activity of allogenic T cells and corresponding quantities of soluble HLA class I molecules in the plasma (Figure 2B). Together, these results indicate that soluble HLA class I isoforms represent a plasma component that decreases stimulatory properties of MDDC during progressive HIV-1 infection.

**Figure 3:**
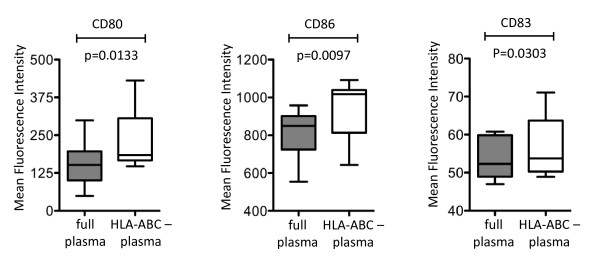
**Soluble HLA class I molecules inhibit surface expression of co-stimulatory molecules on MDDC**. Box and Whisker plots indicate mean fluorescence intensity of indicated costimulatory molecules on MDDC following exposure to full or HLA class I-depleted plasma from untreated HIV-1 patients with progressive disease.

To further investigate immunoregulatory effects of soluble HLA class I molecules on dendritic cells, we analyzed cytokine secretion properties and surface expression of costimulatory molecules of MDDC following exposure to full or HLA class I-depleted plasma from persons with progressive disease. We observed that depletion of HLA class I molecules from plasma resulted in increased expression of the costimulatory molecules CD80, CD86, and CD83 on MDDC; this is consistent with an inhibitory effect of soluble HLA class I molecules on the expression intensity of co-stimulatory molecules on MDDC (Figure 3). In contrast, secretion of TNF-α, IL-6 and IL-12p70 was not different in MDDC exposed either to full or HLA class I depleted plasma (data not shown), indicating that soluble HLA class I isotypes do not play a major role for regulating cytokine secretion characteristics of MDDC.

**Figure 4:**
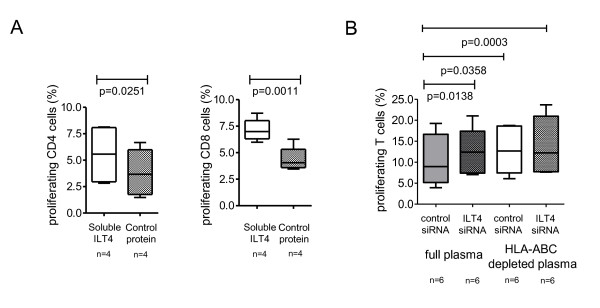
**Soluble HLA class I molecules inhibit functional properties of MDDC through interactions with LILRB2**. (A): Data indicate allostimulatory properties of MDDC after exposure to plasma from HIV-1 progressors in the presence of recombinant soluble LILRB2 or control protein. (B): Allostimulatory properties of MDDC after exposure to HLA class I-deficient or -proficient plasma from progressors in the presence or absence of siRNA-mediated LILRB2 silencing.

To explore mechanisms of how soluble HLA class I isoforms can mechanistically reduce stimulatory properties of MDDC, we focused on their interactions with inhibitory myelomonocytic MHC class I receptors from the LILR family that are constitutively expressed on dendritic cells, and upregulated during chronic progressive infection [5,6]. LILRB2 is a prominent member of the LILR family that can induce powerful suppression of dendritic cells following recognition of membrane-bound [17] and soluble HLA heavy chains [18]; the surface expression of this receptor on dendritic cells is strongly upregulated in persons with progressive or non-progressive HIV-1 infection (Additional File 4). As shown in Figure 4A, blockade of the LILR binding sites within soluble HLA class I molecules by use of soluble recombinant LILRB2 proteins increased functional antigen-presenting properties of MDDC, while a control protein (Gluthatione-S-Transferase) has no similar effects; this suggests that inhibitory effects of soluble HLA class I molecules on MDDC are predominantly mediated by interactions with LILRB2. To further investigate this, we analyzed stimulatory properties of MDDC in the presence or absence of HLA class I-depleted plasma after targeted siRNA-mediated knock-out of LILRB2 in MDDC. Electroporation with LILRB2-specific siRNA highly effectively reduced LILRB2 surface expression on MDDC (Additional File 5). Our experiments demonstrated that following siRNA-mediated downregulation of LILRB2 in MDDC, plasma from HIV-1 progressors was no longer able to reduce the stimulatory effects of MDDC. Moreover, after silencing of LILRB2 expression in MDDC, no difference was detectable between the allostimulatory activities of MDDC exposed to HLA class I-proficient or -deficient plasma from progressors, further indicating that soluble HLA class I molecules inhibit functional antigen-presenting properties of dendritic cells via binding to LILRB2 (Figure 4B).

## Discussion

As the most effective professional antigen-presenting cells, dendritic cells play critical roles for generating, maintaining and fine-tuning T-, B- and NK-cell mediated immune responses against HIV-1. In persons with natural control of HIV-1 infection, myeloid dendritic cells have superior antigen-presenting properties [6], while severe dysfunction of these cells has been reported in advanced stages of HIV-1 infection [17,19]. This suggests that dendritic cell function can importantly influence clinical HIV-1 disease outcomes, but mechanistic pathways regulating the functional activity of these cells in HIV-1 infection remain poorly defined. In the present study, we demonstrate that soluble HLA class I molecules serve as systemic inhibitors of the functional properties of dendritic cells by interacting with inhibitory myelomonocytic MHC class I molecules from the LILR family. This indicates that in addition to their well-documented role for inhibiting various effector cell populations of the immune system [11-13], soluble HLA class I isoforms can contribute to progressive immune dysfunction in advanced HIV-1 infection by inhibiting professional antigen-presenting cells.

Multiple soluble factors have been identified that lead to systemic perturbations of immune system during HIV-1 infection. Most dominantly, such soluble factors include pro-inflammatory cytokines which are secreted in larger amounts by dendritic cells, monocytes, T cells and B cells during progressive HIV-1 infection [20,21]. The source of the elevated levels of soluble HLA class I isoforms in the plasma of persons with progressive HIV-1 infection remains to be determined in detail, but increased intracellular accumulation of HLA class I isoforms in monocytes and dendritic cells suggests an autocrine effect by which release of these molecules from professional antigen-presenting cells autoregulates the functional properties of the secreting cells. Obviously, such a view does not exclude paracrine effects of soluble HLA class I molecules on other cell types localized in the surrounding microenvironment. Moreover, it is likely that soluble HLA class I molecules represent only one of multiple additional plasma components that regulate functional characteristics of dendritic cells; other intact or proteolytic fragments of plasma proteins may also have an important influence on dendritic cell functions, and remained to be investigated in future studies.

In this study, we found that soluble HLA class I molecules inhibit allostimulatory functions of DC through interactions with LILRB2, although cytokine secretion of dendritic cells remained unaffected by interactions between LILRB2 and soluble HLA class I molecules. This contrasts to the effects of soluble HLA-G molecules, which can alter both antigen-presenting properties and secretion of pro-inflammatory cytokines in dendritic cells [22]. Together, these results suggest that individual functional properties of dendritic cells are differentially affected by triggering of LILRB2 through either classical or non-classical soluble HLA class I molecules. Generally, LILRB2 is highly upregulated on DC during chronic progressive HIV-1 infection [5] and seems to play an important role for inducing a tolerogenic profile of dendritic cells in the setting of immune tolerance after solid organ transplantation [23] and during pregnancy [24]. In previous studies, tolerogenic effects of LILRB2 were attributed to interactions with membrane-bound HLA class I molecules; however, the results presented here suggest that binding of soluble HLA class I isotypes can similarly trigger LILRB2-mediated tolerogenic effects in DC. This is well in line with the recent description of strong interactions between LILRB2 and soluble HLA class I heavy chains [18].

Specific HLA class I alleles are among the best predictors of spontaneous HIV-1 disease progression [25,26], and it will be important to determine whether associations between specific HLA class I isoforms and HIV-1 disease outcomes may be at least in part attributable to immunoregulatory effects of soluble HLA class I molecules on dendritic cells. Previous work has indeed shown that dendritic cell dysfunction resulting from interactions between specific membrane-bound HLA class I molecules and MHC class I receptors from the LILR family can critically determine HIV-1 disease progression [18,27]. Moreover, HLA allele-specific interactions between soluble HLA class I heavy chains and immunoregulatory receptors from the LILR family have recently been demonstrated using a bead-based assay in a cell-free system [18]. Future investigations will be necessary to compare functional immunoregulatory effects of individual soluble HLA class I isoforms associated with distinct HIV-1 disease outcomes.

## Conclusions

Our data suggest that high levels of circulating soluble HLA class I molecules during progressive HIV-1 infection contribute to HIV-1-associated immune dysfunction by reducing stimulatory properties of myeloid dendritic cells. In this way, soluble HLA class I molecules may exert important immunoregulatory effects that propagate immune deficiency and viral persistence during HIV-1 infection.

## Material and Methods

### Study subjects

Study participants were recruited at the Massachusetts General Hospital after giving written consent according to protocols approved by the local Institutional Review Board. HIV-1 patients were not taking antiretroviral therapy at the time of study participation.

### Western blots

Equal amounts of plasma were subjected to SDS-PAGE (Tris-Glycine 4-20% gels, Invitrogen), electroblotted and incubated with antibodies specific for HLA class I (clone T25.99 recognizing the HLA class I alpha3 domain [28], kindly provided by Dr. Soldano Ferrone, University of Pittsburg), β2-microglobulin (clone BBM.1, Santa Cruz Biotechnology) or β-actin (Abcam), followed by visualization with HRP-labeled secondary antibodies and ECL detection reactions (Amersham Biosciences).

### Flow cytometric analysis of HLA-ABC expression

PBMCs were incubated with monoclonal antibodies against HLA-A, -B -C isoforms (clone W6/32, specific for HLA class I/β2-microglobulin complexes), CD14, HLA-DR, CD11c and a cocktail of lineage antibodies (CD3, CD14, CD16, CD19, CD56) for 20 min at room temperature. For intracellular detection of HLA-class I isoforms, cells were stained with surface antibodies, fixed and permeabilized using a commercial kit (Caltag), followed by intracellular staining with biotinylated HLA class I-specific antibodies (clone W6/32) and streptavidin-PE. Cells were analyzed on an LSRII (BD Biosciences) instrument.

### Depletion of soluble HLA-ABC

Plasma samples were incubated with biotinylated HLA class I antibody (clone W6/32) for 1 h at room temperature, followed by immunomagnetic depletion of HLA class I molecules using anti-biotin magnetic beads (Invitrogen).

### Preparation of Monocyte-derived dendritic cells (MDDC)

Freshly isolated PBMC were incubated for 60 min at 37°C to adhere monocytes. Adherent monocytes were propagated for 5d in the presence of 50 ng/ml GM-CSF (CellGenix). Immature myelomonocytic cells were then incubated with either full plasma or HLA-class I-depleted plasma at 37°C for 30 min, followed by aggressive washes. In some experiments, recombinant LILRB2 protein (R&D Systems) was added to full plasma for 1 h before adding to the MDDC. When indicated, MDDC were electroporated with LILRB2-specific siRNA as described before [6]. MDDC were matured for 16 h using a previously described cytokine cocktail containing IL-1β, TNF-α, PGE-2, and IL-6 [6].

### Dendritic cell functional assays

Matured MDDC were mixed with CFSE-labeled allogeneic T cells from identical, HIV-1 negative donors. After 6 consecutive days of culture, cells were stained with monoclonal CD4+ and CD8+ antibodies and analyzed by flow cytometry. For the detection of cytokine secretion in MDDC, immatured cells were stimulated with 5 μg/ml TLR7/8 ligands CL097 (InvivoGen, San Diego, CA) for 20 h in the presence of Brefeldin A. After fixation and permeabilization, intracellular cytokine staining was performed using antibodies against IL-6, IL-12p70, TNF-α.

### Statistics

Data were expressed using Box- and Whisker plots, indicating the median, minimum, maximum and the 25^th ^and 75^th ^percentiles. Statistical comparisons were made using Mann Whitney U tests or Wilcoxon rank sum tests, as appropriate.

## Competing interests

The authors declare that they have no competing interests.

## Authors' contributions

JH, MA-A, JR, MC and KS carried out the experiments. JH, MA-A and XGY participated in data analysis and interpretation. FP recruited study cohorts. ML and XGY participated in the study design and wrote the manuscript. All authors read and approved the final manuscript.

### Funding Sources

This work was supported by the National Institutes of Health of the US (AI078799 and ARRA supplement to AI078799 to XGY). MAM is the recipient of a fellowship award from the Dubai Harvard Foundation for Medical Research. ML and XGY are both recipients of the Clinical Scientist Development Award from the Doris Duke Charitable Foundation. This work was supported by the Doris Duke Charitable Foundation Grant # 2009034.

## Supplementary Material

Additional File 1 **Western-blot-based detection of β2-microglobulin in the plasma from HIV-1 progressors, elite controllers and HIV-1 negative persons**. Click here for file

Additional File 2 **Phenotypic characteristics of MDDC used for mixed lymphocyte reactions**. Flow cytometry dot plots indicate the gating strategy used for identifying MDDC, the proportion of MDDC expressing the dendritic cell markers CD11c and HLA-DR and the proportion of MDDC expressing HLA class I molecules. Click here for file

Additional File 3 **Efficacy of antibody-mediated depletion of soluble HLA class I molecules from plasma**. Data demonstrate western blot-based detection of soluble class I molecules from plasma before (left panel) and after (middle panel) antibody-mediated depletion. Right panel reflects western blot of HLA class I molecules isolated from plasma during depletion procedure. Click here for file

Additional File 4 **Surface expression of LILRB2 on peripheral blood dendritic cells in HIV-1 elite controllers, HIV-1 progressors and HIV-1 negative control subjects**. Click here for file

Additional File 5 **Efficacy of si-RNA mediated silencing of LILRB2 in MDDC**. Data indicate flow cytometric detection of LILRB2 on cell surface of MDDC before and after electroporation with LILRB2-specific siRNA or control siRNA. One representative example is shown. Click here for file
