# Supplementary figures and images for: Systemic inhibition of myeloid dendritic cells by circulating HLA class I molecules in HIV-1 infection
Source: Retrovirology. 2012 Jan 30;9:11. doi: 10.1186/1742-4690-9-11 (PMC3308926; doi:10.1186/1742-4690-9-11)

## Slide 1
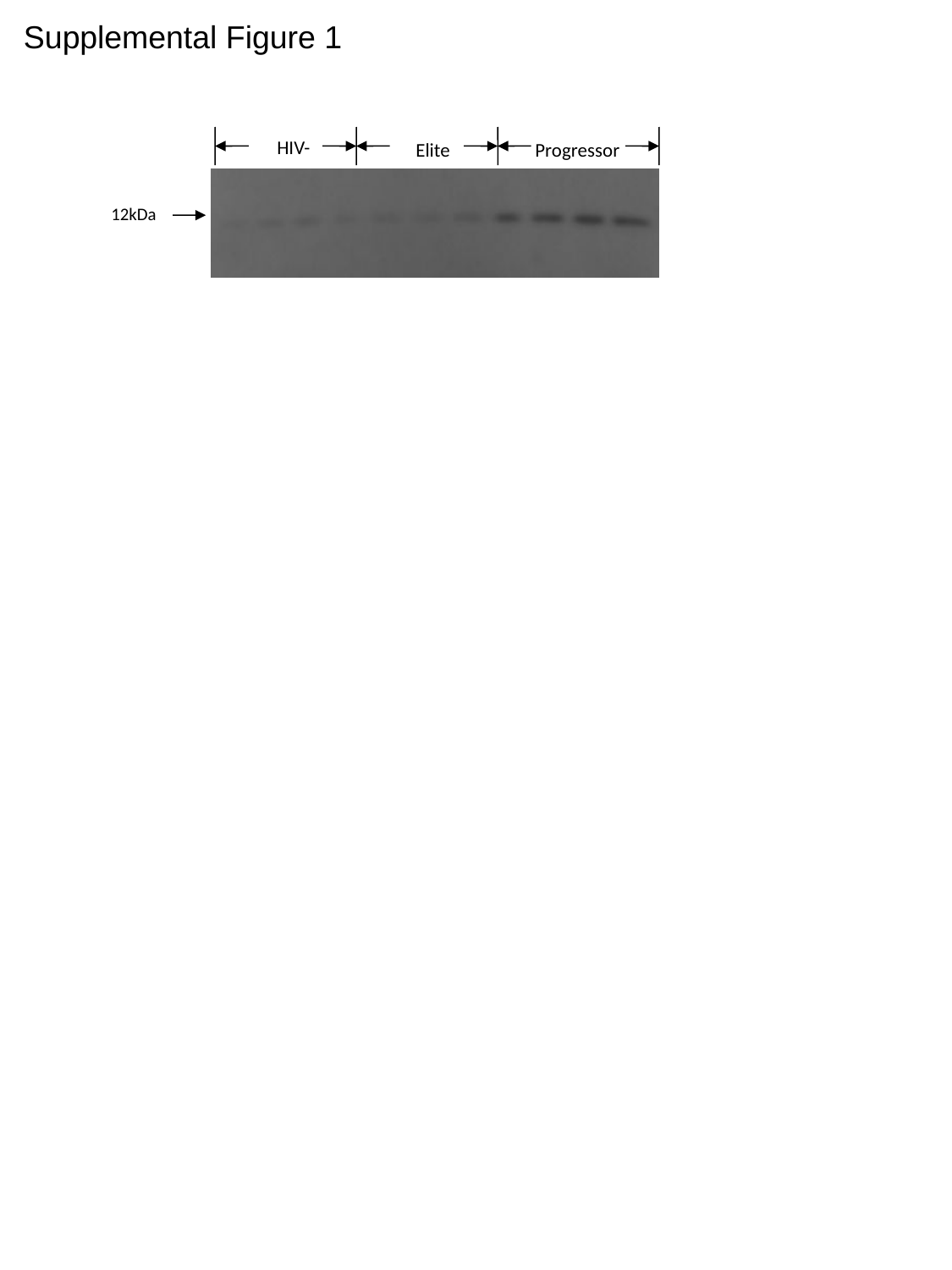

Supplemental Figure 1
HIV-
Elite
Progressor
 12kDa

Supplement: Additional File 1 — Western-blot-based detection of β2-microglobulin in the plasma from HIV-1 progressors, elite controllers and HIV-1 negative persons. [file 1742-4690-9-11-S1.PPT]

## Slide 1
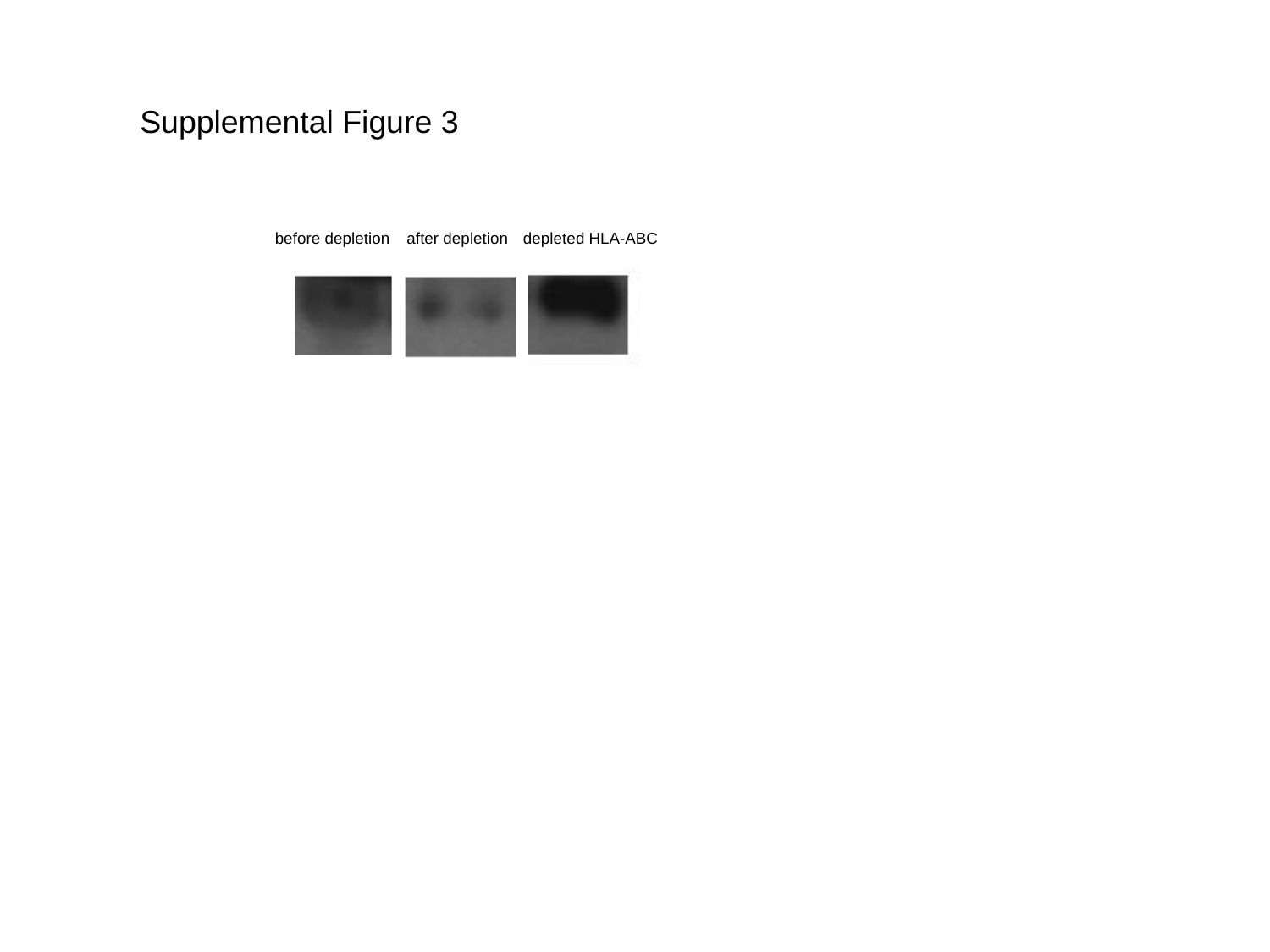

Supplemental Figure 3
before depletion
after depletion
 depleted HLA-ABC

Supplement: Additional File 3 — Efficacy of antibody-mediated depletion of soluble HLA class I molecules from plasma. Data demonstrate western blot-based detection of soluble class I molecules from plasma before (left panel) and after (middle panel) antibody-mediated depletion. Right panel reflects western blot of HLA class I molecules isolated from plasma during depletion procedure. [file 1742-4690-9-11-S3.PPT]

## Slide 1
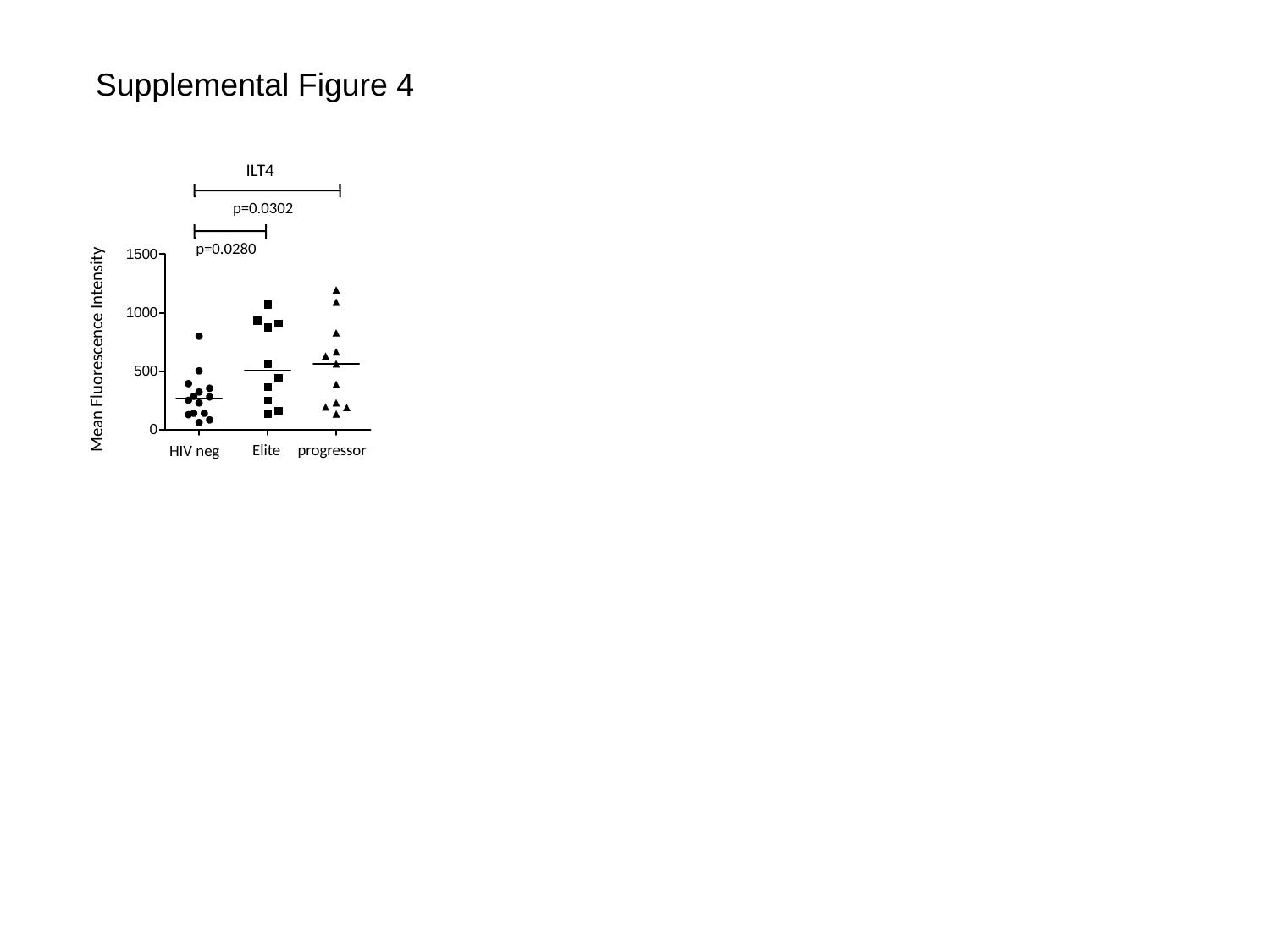

Supplemental Figure 4
ILT4
p=0.0302
p=0.0280
Mean Fluorescence Intensity
Elite
progressor
HIV neg

Supplement: Additional File 4 — Surface expression of LILRB2 on peripheral blood dendritic cells in HIV-1 elite controllers, HIV-1 progressors and HIV-1 negative control subjects. [file 1742-4690-9-11-S4.PPT]
