# Supplementary material for: Systemic inhibition of myeloid dendritic cells by circulating HLA class I molecules in HIV-1 infection
Source: Retrovirology. 2012 Jan 30;9:11. doi: 10.1186/1742-4690-9-11 (PMC3308926; doi:10.1186/1742-4690-9-11)
Supplement: Additional File 2 — Phenotypic characteristics of MDDC used for mixed lymphocyte reactions. Flow cytometry dot plots indicate the gating strategy used for identifying MDDC, the proportion of MDDC expressing the dendritic cell markers CD11c and HLA-DR and the proportion of MDDC expressing HLA class I molecules. [file 1742-4690-9-11-S2.PPT]

## Slide 1
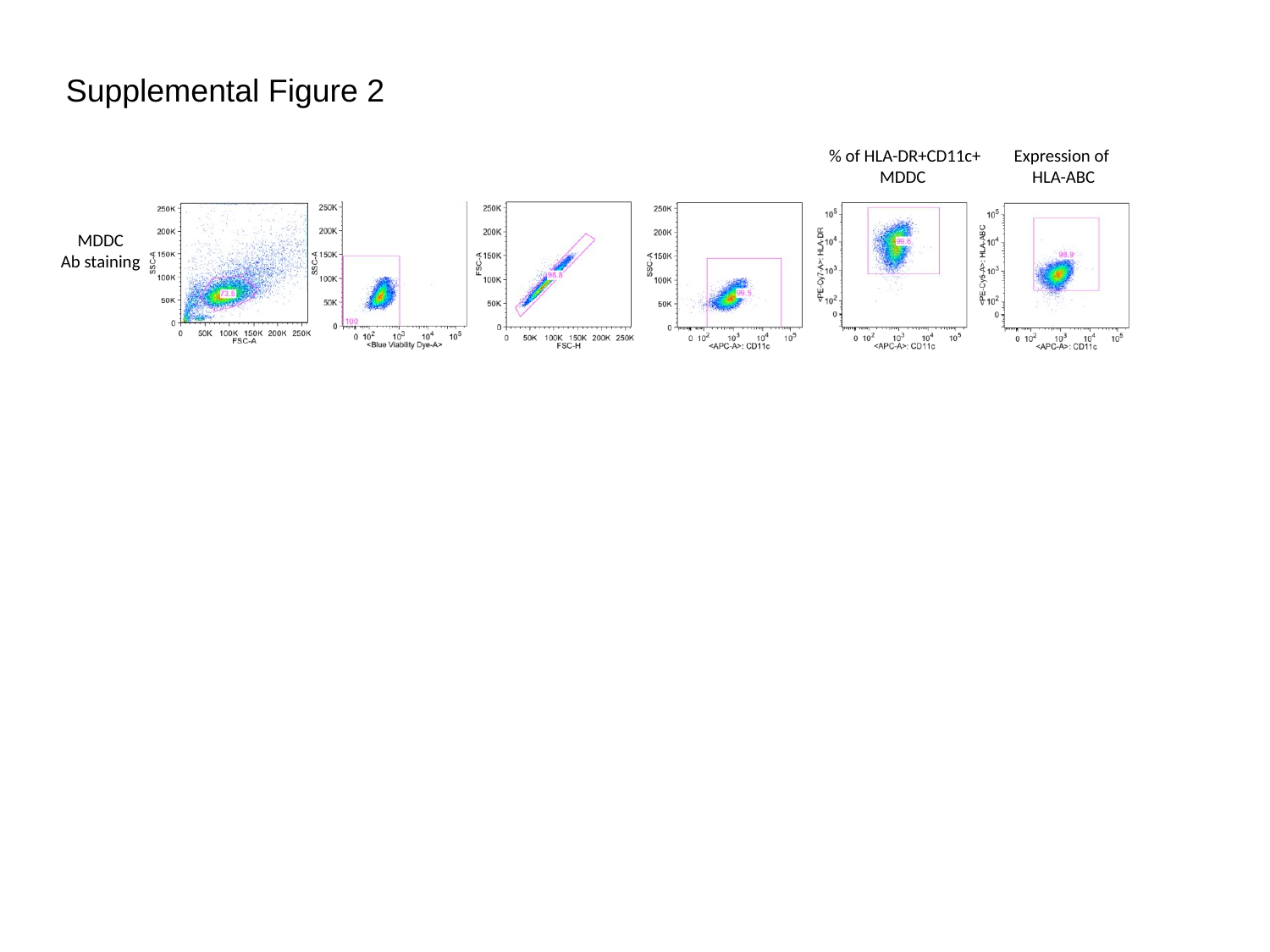

Supplemental Figure 2
% of HLA-DR+CD11c+ MDDC
Expression of
HLA-ABC
MDDC
Ab staining
MDDC
background
