# Supplementary material for: Systemic inhibition of myeloid dendritic cells by circulating HLA class I molecules in HIV-1 infection
Source: Retrovirology. 2012 Jan 30;9:11. doi: 10.1186/1742-4690-9-11 (PMC3308926; doi:10.1186/1742-4690-9-11)
Supplement: Additional File 5 — Efficacy of si-RNA mediated silencing of LILRB2 in MDDC. Data indicate flow cytometric detection of LILRB2 on cell surface of MDDC before and after electroporation with LILRB2-specific siRNA or control siRNA. One representative example is shown. [file 1742-4690-9-11-S5.PPT]

## Slide 1
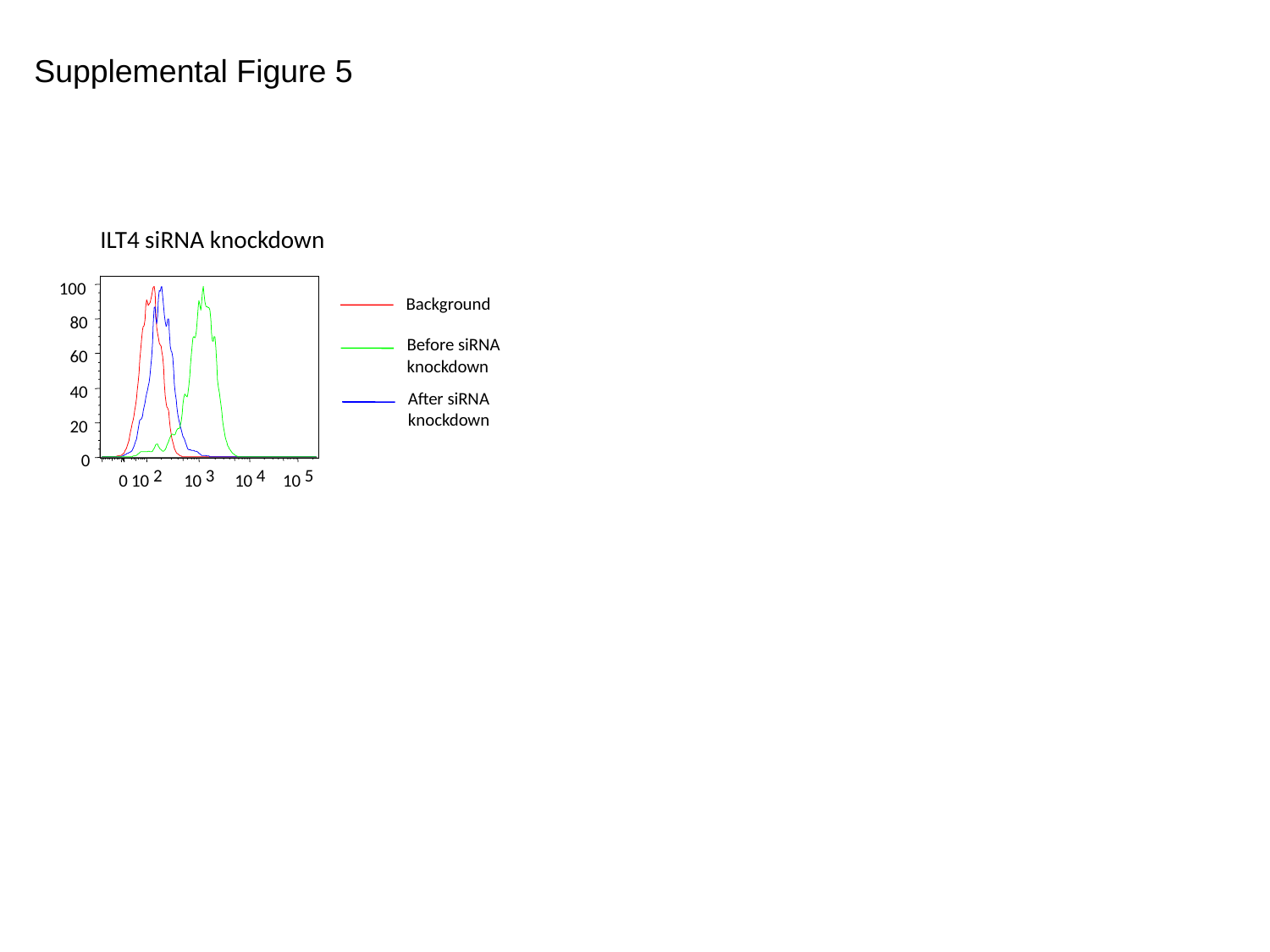

Supplemental Figure 5
ILT4 siRNA knockdown
100
80
60
40
20
0
2
3
4
5
0
10
10
10
10
Background
Before siRNA
knockdown
After siRNA
knockdown
